# Somatic mutations associated with MRI-derived volumetric features in glioblastoma

**DOI:** 10.1007/s00234-015-1576-7

**Published:** 2015-09-04

**Authors:** David A. Gutman, William D. Dunn, Patrick Grossmann, Lee A. D. Cooper, Chad A. Holder, Keith L. Ligon, Brian M. Alexander, Hugo J. W. L. Aerts

**Affiliations:** Departments of Neurology, Emory University School of Medicine, Atlanta, GA USA; Biomedical Informatics, Emory University School of Medicine, 1648 Pierce Dr NE, Atlanta, GA 30307 USA; Radiology and Imaging Sciences, Emory University School of Medicine, Atlanta, GA USA; Department of Biomedical Engineering, Georgia Institute of Technology, Atlanta, GA USA; Department of Radiation Oncology, Dana-Farber Cancer Institute, Brigham and Women’s Hospital, Harvard Medical School, Boston, MA USA; Radiology, Dana-Farber Cancer Institute, Brigham and Women’s Hospital, Harvard Medical School, Boston, MA USA; Pathology, Dana-Farber Cancer Institute, Brigham and Women’s Hospital, Harvard Medical School, Boston, MA USA

**Keywords:** Radiogenomics, GBM, MRI, Imaging genomics, Volumetrics

## Abstract

**Introduction:**

MR imaging can noninvasively visualize tumor phenotype characteristics at the macroscopic level. Here, we investigated whether somatic mutations are associated with and can be predicted by MRI-derived tumor imaging features of glioblastoma (GBM).

**Methods:**

Seventy-six GBM patients were identified from The Cancer Imaging Archive for whom preoperative T1-contrast (T1C) and T2-FLAIR MR images were available. For each tumor, a set of volumetric imaging features and their ratios were measured, including necrosis, contrast enhancing, and edema volumes. Imaging genomics analysis assessed the association of these features with mutation status of nine genes frequently altered in adult GBM. Finally, area under the curve (AUC) analysis was conducted to evaluate the predictive performance of imaging features for mutational status.

**Results:**

Our results demonstrate that MR imaging features are strongly associated with mutation status. For example, TP53-mutated tumors had significantly smaller contrast enhancing and necrosis volumes (*p* = 0.012 and 0.017, respectively) and RB1-mutated tumors had significantly smaller edema volumes (*p* = 0.015) compared to wild-type tumors. MRI volumetric features were also found to significantly predict mutational status. For example, AUC analysis results indicated that TP53, RB1, NF1, EGFR, and PDGFRA mutations could each be significantly predicted by at least one imaging feature.

**Conclusion:**

MRI-derived volumetric features are significantly associated with and predictive of several cancer-relevant, drug-targetable DNA mutations in glioblastoma. These results may shed insight into unique growth characteristics of individual tumors at the macroscopic level resulting from molecular events as well as increase the use of noninvasive imaging in personalized medicine.

**Electronic supplementary material:**

The online version of this article (doi:10.1007/s00234-015-1576-7) contains supplementary material, which is available to authorized users.

## Introduction

Glioblastoma (GBM) is the most common and most aggressive form of brain cancer with a median survival of less than 15 months and a 5-year survival rate of less than 10 % [1]. While factors ranging from younger age at diagnosis, cerebellar location, better cognitive performance, and more extensive tumor resection have been associated with more favorable outcome, the current standard of care treatment involving surgery, radiation, and chemotherapy ultimately fails, in part due to the proliferative and diffusely infiltrative nature of the tumor [2]. Recent molecular analyses have demonstrated significant diversity in histologically similar tumors that drive proliferation and competitive propagation [3]. In addition, integrated analyses using gene expression, copy number, methylation, and somatic mutation patterns have identified distinct GBM subtypes, some of which associated with distinct responses to treatment [4].

Methods such as magnetic resonance imaging (MRI) that can noninvasively characterize the tumor at a macroscopic scale can be of potential value, as they can provide complementary information to the tumor’s molecular characterization [5]. Historically, only very basic parameters have been derived from imaging data, such as measurements of tumor size based on “representative” cross sections on a single radiology image [6–8]. While such measures are easy to perform and serve as the basis for assessing treatment response [9, 10], there is a rich set of additional visual characteristics of the tumor that can also be assessed. One effort to catalog these characteristics is the VASARI Research Project, which seeks to develop a controlled vocabulary describing the varied morphology of glioblastoma (http://cabig.cancer.gov/action/collaborations/vasari/). The VASARI feature set was developed by The Cancer Genome Atlas (TCGA) radiology working group and uses a standard lexicon with the goal of reproducibly assessing 26 imaging descriptors based on T1-weighted and T2-weighted FLAIR MRI modalities. For example, variables such as major axis length, tumor location, proportion enhancing, thickness of enhancing margin, and proportion of edema are all measured by trained radiologists in this protocol. Data obtained from this protocol has led to a number of findings demonstrating the value of adding imaging data to models predicting survival [11, 12] and molecular profile [13] in glioblastoma.

Our current work expands upon these results by using a semi-automated digital quantification technique, which recent work has shown to be more objective and lead to more robust findings than qualitative staging methods used in the past [14]. Indeed, feature measurements based on manual estimations have been shown to be subject to substantial inter- and intra-observer variability [15]. Recently, quantitative volumetric measurements of tumor subvolumes, or imaging features, such as contrast-enhancing tumor, necrosis, and tumor-associated edema, have been associated with response to treatment and overall prognosis [16–18]. Associations of tumor subvolume data and somatic mutations would be of clinical importance, as it would improve our understanding and macroscopic implications of these heavily researched molecular events [19, 20].

In this study, we investigated whether somatic mutations in genes consistently implicated in glioblastoma are associated with and can be predicted by digitally derived volumetric features from tumor MR images, including contrast enhancing, necrosis, and T2 FLAIR hyperintensity volumes, as well as combination and ratios of these features. We chose to focus on somatic mutations due to the strong literature presence and clinical relevance. We limited our analysis to an a priori selected gene set as described by Verhaak et al. [4], as well as showing mutations in at least five patients included in our cohort, resulting in the following: TP53, PTEN, NF1, EGFR, IDH1, PIK3R1, RB1, PIK3CA, and PDGFRA. Many of these genes are drug-targetable, raising the possibility of treating cancer based on noninvasively derived imaging biomarkers. In particular, several potential therapies to target mutant EGFR such as monoclonal antibodies, vaccines, or small molecule inhibitors are currently being actively investigated [21]. In addition, some PTEN nonsense mutations have shown to be targeted by drugs that inhibit PKC (byrostatin) and Raf (AZ628) [22], and IDH1 mutations have been shown to be targeted by small molecule inhibitors such as AGI-120 [23]. Noninvasive methods that can predict mutation status would thus be of great clinical importance and could guide clinical treatment decision-making, especially in situations where molecular testing or surgical biopsy is not feasible or appropriate.

## Materials and methods

### Imaging and mutation data

Presurgical T1-weighted post-Gd contrast (T1C) and T2-weighted FLAIR sequence MR images were downloaded from The Cancer Imaging Archine (TCIA) (http://thecancerimagingarchive.net) in September of 2014. TCIA is an NCI-sponsored imaging sharing resource that houses more than three million images from 31 different institutions [24, 25]. This resource is particularly valuable because it stores a wide variety of publically available longitudinal data with associated genomic data, which is not readily obtainable at a single institution level. As the patients had been previously de-identified by TCGA and were available for public download, no Institutional Review Board approval was required. Since presurgical status of an image was not explicitly included in the TCIA data, presurgical status was verified by a trained neurologist (CH, 17 years of experience). Somatic mutation status from whole exome sequencing and clinical data were downloaded from TCGA using cBioPortal (http://www.cbioportal.org/public-portal/) queried with the “cgdsr” package version 1.1.30 in R [26]. The latest cBioPortal GBM dataset (version “provisional”) was downloaded on September 25, 2014. Within TCIA, there were 185 GBM patients with both T1C and T2-weighted FLAIR images available before surgery. Of these, 76 patients had mutation data available within TCGA and were included in this analysis.

### Volumetric image analysis

For T1C images, 2D masks were drawn on each MRI slice over the visible tumor using FSLView, a module in the FMRIB Software Library 5.0 (FSL [27]). For these image sets, a single contour encapsulating both the dark (necrosis) and bright (contrast enhancing) areas was segmented, becoming the basis of what will be referred to as tumor bulk volume. Similarly, for the T2-weighted FLAIR image sets, a single contour was drawn on each slice over the visible tumor which encompassed both the region previously identified on the T1C (i.e., tumor bulk) as well as surrounding hyperintense signal including the edema envelope. To note, this markup does not attempt to differentiate between nonenhancing tumor and true edema, as they both appear hyperintense on the FLAIR image.

Following segmentation, the original mask containing the tumor region on the T1C images underwent K-means clustering using the FSL FAST tool (FMRIB’s Automated Segmentation Tool) [28] to differentiate dark (necrosis) and bright (contrast enhancing) areas from one another. A subset of 5–10 machine-generated segmentations were reviewed by trained experts (CH, DG) to verify proper segmentation; we determined that the FAST algorithm module produced robust segmentations without extensive parameter optimization. This algorithm has been used routinely for more than 10 years to segment white and gray matter, and we found robust segmentation results when we applied the algorithm to segmenting “bright” versus “dark” pixels for contrast enhancement and necrosis.

**Fig. 1:**
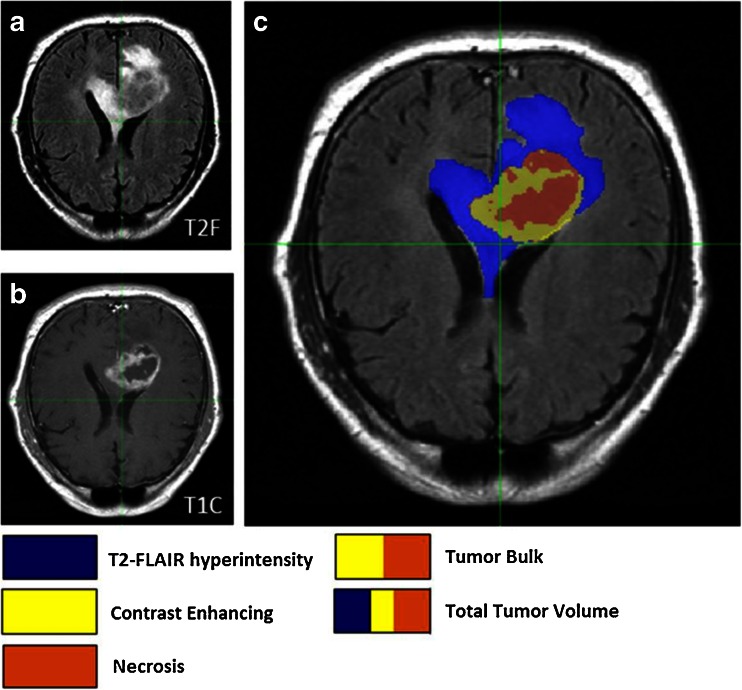
Visualization of naming conventions for the tumor volumetric features used throughout this article for TCGA-02-0033, a 54-year-old male glioblastoma patient. **a** Tumor-associated T2-FLAIR hyperintensity and total tumor volume was quantified from the T2-weighted FLAIR images. **b** Necrosis, contrast enhancing, and tumor bulk volume was quantified from T1-weighted post-Gd contrast (T1C) images. **c** This panel displays both images co-registered and overlayed on top of each other for visualization purposes. Tumor bulk is defined as the total abnormal tumor area on the T1C images: combination of contrast enhancing and necrosis volumes. Total tumor volume is defined as the combination of the tumor bulk and T2-FLAIR hyperintensity volumes

Based on the annotations on the T1C images, contrast enhancing, necrosis, and tumor bulk volumes could be calculated (Fig. 1). In addition, incorporation of the T2-weighted FLAIR series allowed quantification of the total tumor volume and T2-FLAIR hyperintensity volume (total tumor volume–tumor bulk volume). For the purposes of our analyses, several derivative ratios were also calculated: necrosis/contrast enhancing, contrast enhancing/tumor bulk, contrast enhancing/total tumor, necrosis/total tumor, T2-FLAIR hyperintensity/total tumor, and tumor bulk/total tumor volumes. Individual imaging feature volumes were computed by computing the total number of voxels within the respective region and multiplying by the voxel size; these calculations were performed using FSL’s fslstats module.

### Imaging-genomic analysis

For each of the 11 volumes or ratios defined above, the mean values corresponding to patients with mutated genes were compared to those of the wild-type cohort. Significant differences between groups with and without mutations were tested with a two-sided Student’s *t* test. Significance was defined by *p* value <0.05. Normal assumption of volumes was confirmed by *p* value <0.05 under a Shapiro-Wilk test across all patients. This analysis was limited to genes from the a priori selected gene set discussed above.

Predictive power of volumes to predict mutation status was assessed by the area under the curve (AUC) of the receiver operator characteristic (ROC) [29]. To make performance evaluation comparable, we calculated the absolute AUC defined as 0.5 + abs(*x* − 0.5), where *x* is an AUC value. All statistical analysis was carried out by the R statistical software version 3.0.2 on a Linux platform [30].

## Results

To investigate whether somatic mutations were associated with MRI imaging features, the GBM patients analyzed in our analysis were limited to those with mutation data available from TCGA and image data from TCIA (*N* = 76). The genes analyzed in our study were limited to an a priori selected gene set as described by Verhaak et al. [4], as well as showing mutations in at least five patients included in our cohort. This resulted in a total of nine genes included in our analysis: TP53, PTEN, NF1, EGFR, IDH1, PIK3R1, RB1, PIK3CA, and PDGFRA.

### Associations between MRI-derived GBM tumor volumes

**Fig. 2:**
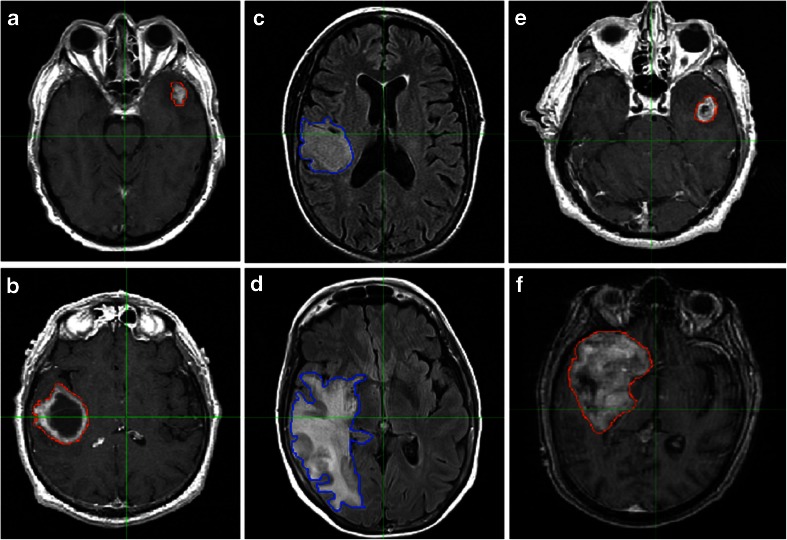
Examples of images characterized by various imaging features. Representative scans of low (**a**) and high (**b**) necrosis/total tumor volume ratios, low (**c**) and high (**d**) FLAIR/total tumor volume ratios, and low (**e**) and high (**f**) tumor bulk volumes are visualized. Masks outline areas used to determine various volumetric features used throughout the project (*red* for tumor bulk on T1-weighted post-Gd contrast (T1C) images, *blue* for T2-FLAIR hyperintensity on T2-weighted FLAIR images)

An initial visual inspection of segmented images demonstrated a wide variety of measured features across patients that could be capitalized upon in imaging genomic analyses (Fig. 2).

**Fig. 3:**
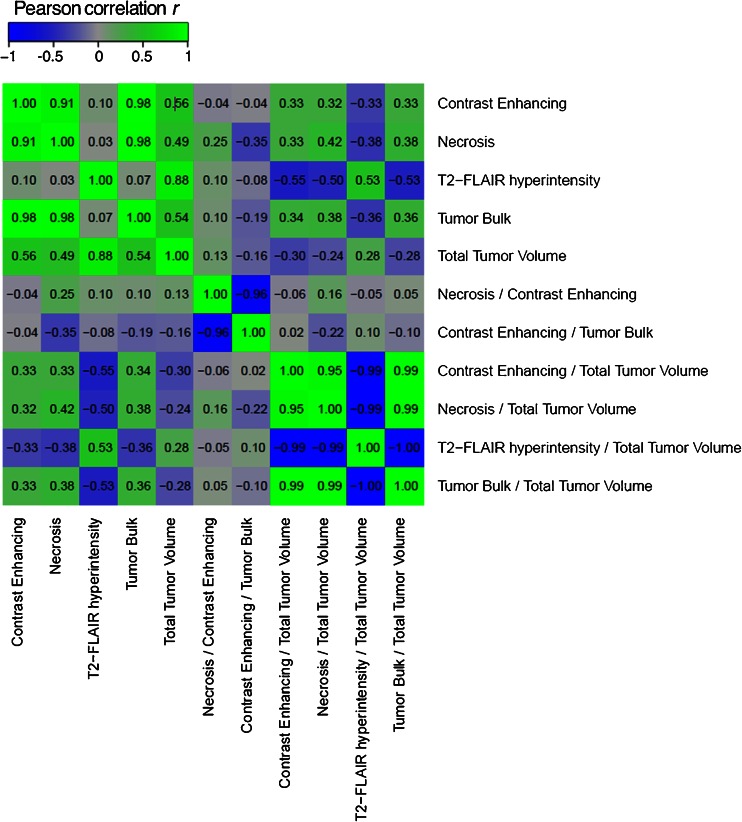
Correlation coefficient matrix between the eleven imaging feature measurements used in this study. Note the high correlation between several features (e.g., T2-FLAIR hyperintensity and total tumor volume), as well as the low correlation between other features (e.g., necrosis and T2-FLAIR hyperintensity). Correlations were assessed using Pearson correlation coefficient

The pairwise Pearson correlation coefficients of these 11 analyzed imaging features are shown in Fig. 3. These results demonstrated the relative independence of many of these volumetric features and ratios. While some higher correlations were noted (e.g., contrast enhancement and necrosis, *r* = 0.91, or T2-FLAIR hyperintensity and total tumor volume, *r* = 0.87), several features also showed low correlations, indicating independence (e.g., necrosis and T2-FLAIR hyperintensity, *r* = 0.07).

**Fig. 4:**
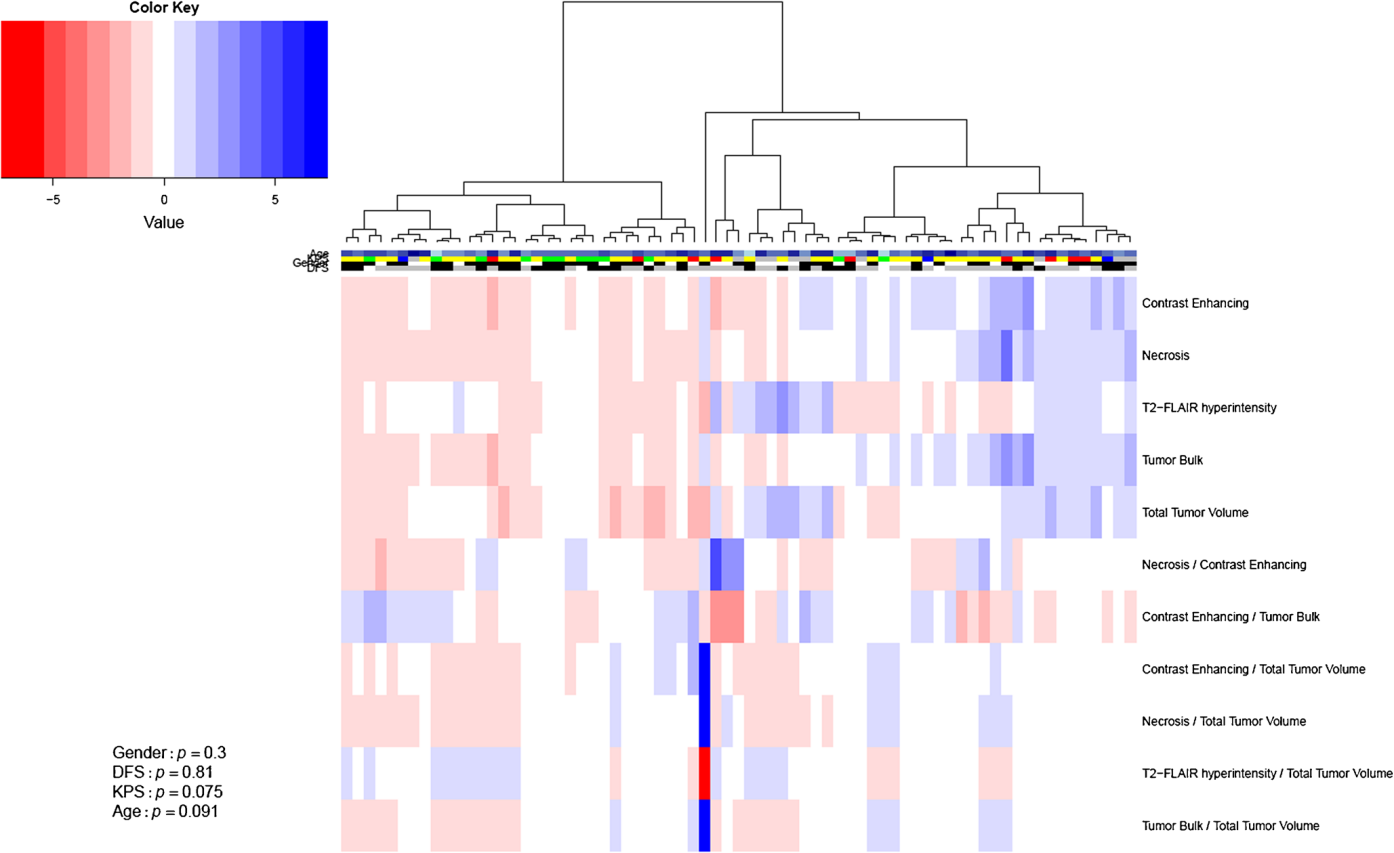
Heatmap of volume values (Z-scores) and clinical parameters. Patients are clustered according to their imaging features (*rows*). The two main clusters show no significant association (chi-squared test) to clinical parameters gender, disease-free status (DFS), Karnofsky performance score (KPS), and age. KPS of 40, 60, 80, and 100 are indicated by *blue*, *red*, *yellow*, and *green*, respectively. Age is indicated by one darker nuance every 10 years (range 21 to 85 years). *Gray* bars indicate clinical parameters that were not available for a patient

In a final exploratory analysis, patients were clustered into two groups based on imaging volumes. Importantly, chi-squared tests did not indicate any significant differences of gender, disease-free status, Karnofsky performance score, and age between these groups, suggesting that the measured imaging volumes measured are relatively independent of these clinical variables (Fig. 4).

### Association of MRI volumetric features with somatic mutations

**Fig. 5:**
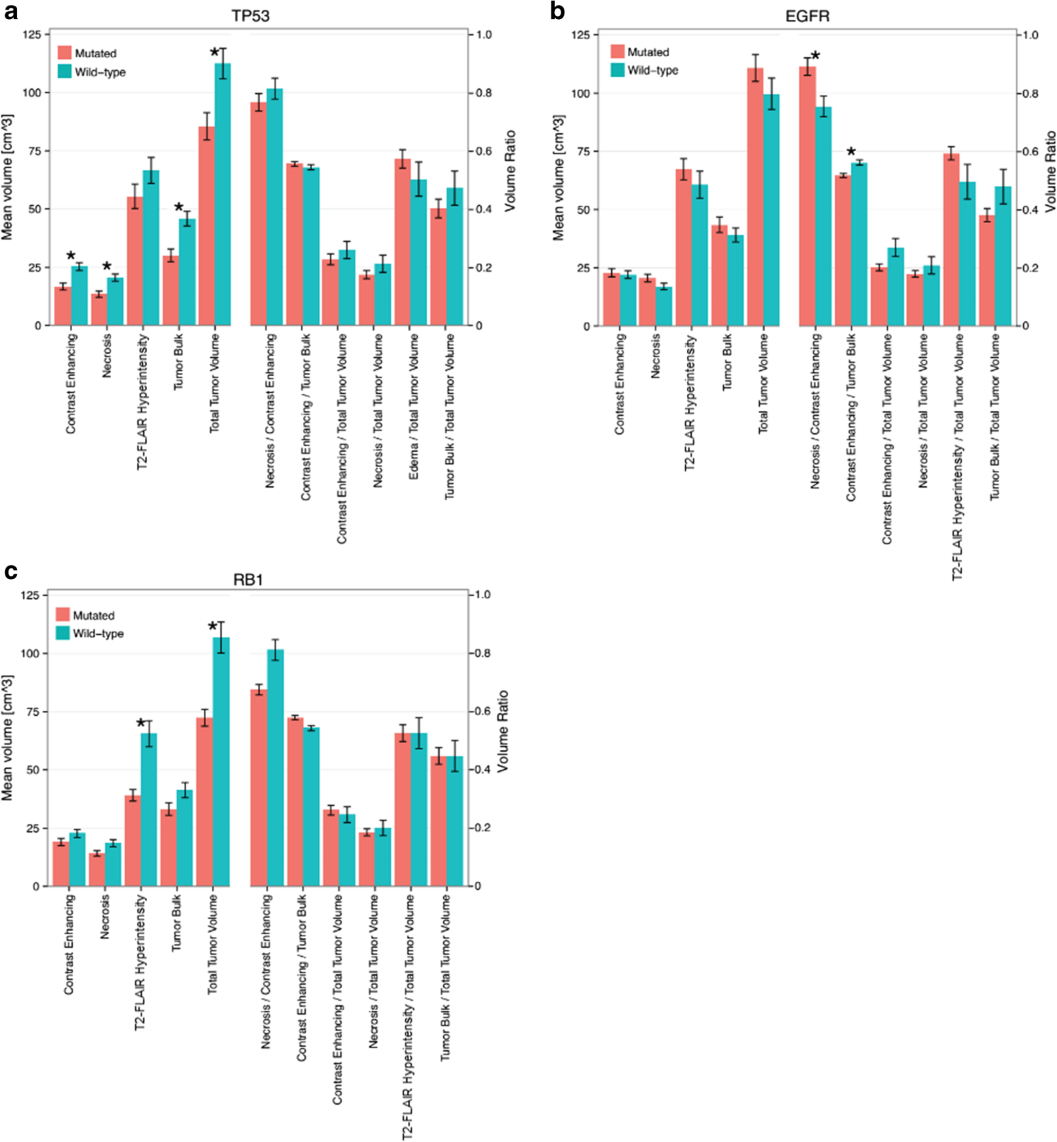
Volumetric differences for mutated versus wild-type tumors for **a** TP53, **b** EGFR, and **c** RB1. For each plot, the left y-axis corresponds to the mean volume of the left features and the right y-axis corresponds to the volume ratio of the features on the right. The bars indicate the standard error of the mean. Note TP53-mutated tumors were found to have significantly smaller contrast enhancing, necrosis, and tumor bulk volumes compared to wild type. EGFR-mutated tumors have a significantly larger necrosis/contrast enhancing ratio, as well as a significantly smaller contrast enhancing/tumor bulk ratio. RB1-mutated tumors have significantly larger T2-FLAIR hyperintensity and total tumor volumes, compared to wild-type tumors

In comparing the volumetric averages for the 11 measures between mutant versus wild-type tumors to investigate the associations between mutation status and volumetric features, several significant results were observed (Table 1). TP53-mutated tumors had four subvolumes that were significantly different from wild-type tumors (Fig. 5a). For example, contrast enhancing and necrosis volumes were significantly smaller for mutated tumors (8588.1 mm^3^ average difference (*p* = 0.012) and 7159.2 mm^3^ average difference (*p* = 0.017), respectively). EGFR-mutated tumors showed a significantly higher necrosis/contrast enhancing ratio (*p* = 0.05) and a significantly lower contrast enhancing/tumor bulk ratio (*p* = 0.008) (Fig. 5b). Furthermore, RB1-mutated tumors showed significantly smaller T2-FLAIR hyperintensity (26,354.4 mm^3^ average difference, *p* = 0.015) and total tumor volume (34,467.2 mm^3^ average difference, *p* = 0.020) (Fig. 5c). For the other mutations, the volumetric features did not significantly differ between mutated and wild-type tumors (Table 1, Supplemental Digital Content).

**Table 1:** Differences in imaging feature volumes between mutated and wild-type tumors for a subset of significant results

	Gene:	TP53	EGFR	RB1
	Number of mutations:	26	24	8
Contrast enhancement	Mut–WT difference	−8588.05	818.21	−3676.05
	*t* Test *p* value	0.012*	0.827	0.485
Necrosis	Mut–WT difference	−7159.16	3555.12	−4436.73
	*t* Test *p* value	0.017*	0.303	0.289
T2-FLAIR hyperintensity	Mut–WT difference	−11164.7	6655.63	−26354.4
	*t* Test *p* value	0.326	0.538	0.015*
Tumor bulk	Mut–WT difference	−15747.2	4373.33	−8112.78
	*t* Test *p* value	0.012*	0.533	0.387
Total tumor	Mut–WT difference	−26911.9	11028.92	−34467.2
	*t* Test *p* value	0.04*	0.402	0.02*
Necrosis/contrast enhancement	Mut–WT difference	−0.69	0.14	−0.14
	*t* Test *p* value	0.492	0.05*	0.056
Contrast enhancement/tumor bulk	Mut–WT difference	0.012	−0.046	0.038
	*t* Test *p* value	0.515	0.008*	0.145

“Mut-WT” refers to difference in average between mutated and wild-type groups for the various volumes (in mm^3^) as well as differences in ratios. For each gene and imaging feature, a two-sided Student’s *t* test was performed to measure significance of the difference and the corresponding *p* value is also provided. For complete set of significances in volumetric differences by mutation status, see Table 1 Supplemental Digital Content

*Statistical significance (*p* < 0.05)

### Predicting somatic mutation based on MRI volumetric features

**Fig. 6:**
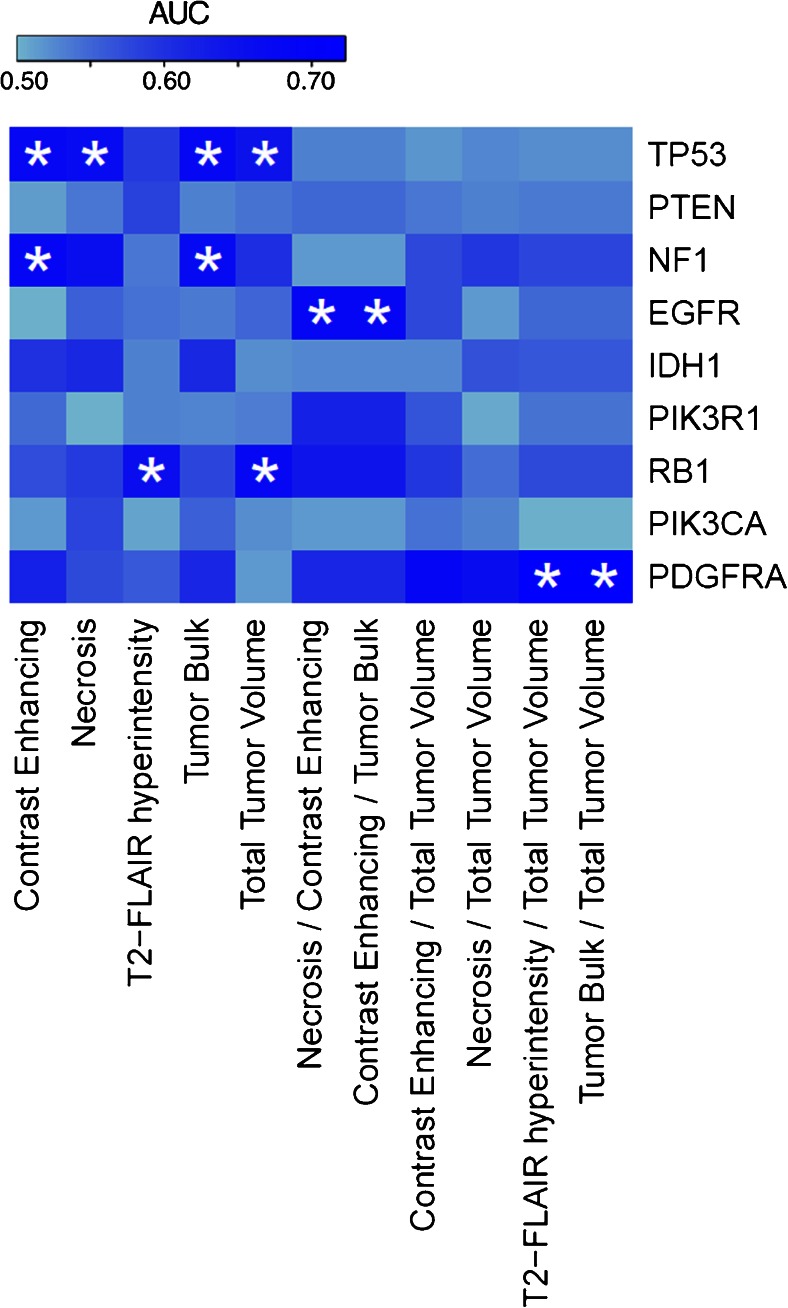
AUC value heatmap. For genes that were mutated in at least five patients, we tested whether the volumes can significantly predict mutation status better than random (*p* < 0.05, *asterisk*), using the area under the curve (AUC) of the receiver operator characteristic (ROC). Volumetric features could significantly predict five out of nine tested mutated genes. We note a tendency for tumor features to predict mutation status of one gene specifically

To assess the potential of the MRI volumetric features to predict somatic mutation status noninvasively, we evaluated the predictive power using the AUC of the receiver-operating characteristic. AUC values were calculated by quantifying the performance of distinguishing between a mutated and wild-type tumor on the basis of each of the 11 imaging features (Fig. 6, Table 2, Table 2 Supplemental Digital Content).

**Table 2:** Gene mutation/volumetric imaging feature correlations for subset containing significant results

	Gene:	TP53	NF1	EGFR	RB1	PDGFRA
	Number of mutations:	26	9	24	8	6
Contrast enhancement	AUC (*p* value)	0.679 (0.001*)	0.681 (0.023*)	0.503 (0.971)	0.57 (0.489)	0.621 (0.258)
	95 % CI	0.569–0.788	0.525–0.837	0.361–0.644	0.372–0.768	0.411–0.831
Necrosis	AUC (*p* value)	0.666 (0.004*)	0.658 (0.063)	0.556 (0.429)	0.588 (0.365)	0.572 (0.531)
	95 % CI	0.552–0.78	0.491–0.825	0.417–0.695	0.397–0.779	0.347–0.796
T2-FLAIR hyperintensity	AUC (*p* value)	0.591 (0.127)	0.537 (0.479)	0.542 (0.499)	0.66 (0.022*)	0.56 (0.687)
	95 % CI	0.474–0.708	0.435–0.639	0.42–0.665	0.523–0.797	0.268–0.852
Tumor bulk	AUC (*p* value)	0.675 (0.002*)	0.671 (0.032*)	0.535 (0.631)	0.577 (0.441)	0.612 (0.322)
	95 % CI	0.566–0.785	0.514–0.828	0.393–0.676	0.381–0.773	0.39–0.833
Total tumor	AUC (*p* value)	0.646 (0.010*)	0.604 (0.103)	0.551 (0.445)	0.676 (0.011*)	0.515 (0.919)
	95 % CI	0.534–0.758	0.479–0.729	0.421–0.681	0.54–0.813	0.218–0.813
Necrosis/contrast enhancement	AUC (*p* value)	0.531 (0.655)	0.516 (0.855)	0.682 (0.001*)	0.642 (0.066)	0.612 (0.375)
	95 % CI	0.397–0.664	0.348–0.683	0.571–0.793	0.491–0.793	0.364–0.859
Contrast enhancement/tumor bulk	AUC (*p* value)	0.531 (0.655)	0.516 (0.855)	0.682 (0.001*)	0.642 (0.066)	0.612 (0.375)
	95 % CI	0.397–0.664	0.348–0.683	0.571–0.793	0.491–0.793	0.364–0.859
T2-FLAIR hyperintensity/total tumor	AUC (*p* value)	0.522 (0.755)	0.576 (0.256)	0.549 (0.488)	0.572 (0.526)	0.722 (0.026*)
	95 % CI	0.383–0.661	0.445–0.707	0.422–0.676	0.35–0.793	0.527–0.918
Tumor bulk/total tumor	AUC (*p* value)	0.522 (0.755)	0.576 (0.256)	0.549 (0.488)	0.572 (0.526)	0.722 (0.026*)
	95 % CI	0.383–0.661	0.445–0.707	0.422–0.676	0.35–0.793	0.527–0.918

For each gene and imaging feature, area under the curve (AUC) values, corresponding *p* value, and 95 % confidence interval are provided. For complete set of correlations, see Table 2 Supplemental Digital Content

*Statistical significance (*p* < 0.05)

In general, we found a tendency for volumetric features to predict mutation status of one gene specifically. For example TP53 could be significantly predicted by contrast enhancing (AUC = 0.68, *p* = 0.001), necrosis (AUC = 0.67, *p* = 0.039), as well as total tumor volumes (AUC = 0.646, *p* = 0.010). Of note, these three volumes were all highly correlated with each other as demonstrated in Fig. 3. Additionally, NF1 mutation status could be predicted by contrast enhancing volume (AUC = 0.68, *p* = 0.023) and tumor bulk volume (AUC = 0.67, *p* = 0.032). EGFR mutations could be predicted by the necrosis/contrast enhancing (AUC = 0.68, *p* = 0.001) ratio and contrast enhancing/tumor bulk (AUC = 0.68, *p* = 0.001) ratio. RB1 mutations could be predicted by T2-FLAIR hyperintensity (AUC = 0.66, *p* = 0.022) and total tumor volume (AUC = 0.68, *p* = 0.011). Finally, PDGFRA could be predicted by T2-FLAIR hyperintensity/total tumor volume (AUC = 0.72, *p* = 0.026) and tumor bulk/total tumor volume ratios (AUC = 0.72, *p* = 0.026). All significant results are summarized in Table 2.

## Discussion

Medical imaging has strong potential to stratify patients, as it is uniquely situated to noninvasively provide a macroscopic evaluation of the entire tumor volume. We quantified GBM MRI phenotypes by defining volumetric features, such as contrast enhancing, necrosis, and T2-FLAIR hyperintensity volumes, and ratios thereof. In this work, we investigated whether quantitative assessments of tumor features are significantly associated with or predictive of somatic mutation status in GBM.

We found strong associations of MRI characteristics with underlying somatic mutation patterns, such as tumor bulk and total tumor volume with TP53 and RB1 mutations. Importantly, these features were able to significantly predict mutations, such as TP53, EGFR, RB1, NF1, and PDGFRA—mutations of clinical importance in GBM [4, 31]. Although the predictive capability of the volumetric features for mutational status was not perfect (i.e., AUC = 1), performance is much higher and significantly different compared to chance (i.e., AUC = 0.50, *p* value ≥ 0.05), demonstrating the strong association of the imaging feature with the underlying driving biology.

Our results showed that contrast enhancing volume and necrosis volume are significantly smaller for TP53 mutants, a finding likely driven by the fact that these tumors, in previous work using the categorically defined VASARI imaging features, have been shown to be characterized by smaller volumes in general [13]. We confirmed this qualitative assessment using our digitally defined quantitative volumetric approach, and additionally showed that both the tumor bulk (on the T1C images) and total tumor volume (on the T2-weighted FLAIR images), were significantly smaller for TP53 mutants.

Our results also show that the necrosis/contrast enhancing ratio was significantly higher in EGFR mutants. These results indicate that although the total tumor volume is similar, EGFR mutants have larger necrosis volumes and smaller contrast enhancing volumes, compared to wild-type tumors. Although the tumor volume was higher for EGFR mutants in our quantitative analysis, the differences were not significant, as previous work had demonstrated [13].

Finally, RB1 mutants were noted to have smaller T2-FLAIR hyperintensity and total tumor volumes but similar contrast enhancing and necrosis volumes compared to wild-type tumors, demonstrating the effect of RB1 on tumor-associated T2-FLAIR hyperintensity. One interpretation of these findings is that these mutations drive different growth patterns within individual tumors that are reflected in drastic differences in the imaged tumor phenotype (for example, highly necrosis/low CE vs. high CE/low necrosis). Correlations between our imaging features demonstrated that in general, the 11 features originally derived from MRI volumes are relatively independent measures of brain tumors characteristics that many have the potential to offer unique insight into tumor behavior (Fig. 3). This was also demonstrated by showing that different features predicted different mutations.

Several automatic and semi-automatic volumetric algorithms have been proposed to segment GBM tumors in relevant subvolumes [32]. For this work, we attempted a novel hybrid approach where we used a trained rater to mask the gross abnormal signal on the T1C and T2-weighted FLAIR image. The gross tumor volume on T1C is then stratified automatically into contrast enhancing and necrosis subvolumes (bright/dark pixels), using the FAST algorithm [33], improving the robustness of the segmentation process. We should note, however, that the segmentations were subsequently manually reviewed at various stages to ensure quality control.

Previous studies have investigated whether different biological subtypes confer different macroscopic properties to the images themselves and significant correlations between genetic expression and macroscopic imaging properties have been established [34–36]. Imaging genomics seeks to close the gap between genomics and neuroradiology to provide a comprehensive quantification of the tumor phenotype by applying a large number of automated image characterization algorithms [14, 20, 37]. In this paper, we applied a relatively low-dimensional feature extraction (focusing on three key volumes and derivatives for eleven features total). Future studies will expand these features and investigate the value of imaging genomics for the prediction of additional mutational patterns.

Going forward, the development of noninvasive imaging biomarkers will provide valuable insight to the clinicians to help in treatment selection and prognosis. As these biomarkers assess the entire tumor volume, they alleviate some of the concerns related to most tissue-based assessments that involve sampling only a small region of the tumor. Given the marked heterogeneity observed within tumor samples taken from even the same patient [38], a noninvasive technique that allows serial imaging (e.g., MRI) can provide valuable insight. Indeed, a limitation to our study is that in our patient set, TCGA tissue sampling was not performed under image guidance and therefore exact location of biopsy is not known. Future studies will investigate the association of intratumor mutational heterogeneity and MRI volumetric features.

In addition, it is important to note that since the TCIA imaging data used in our study was collected through a consortium of several institutions around the country, the specific MR parameters (field strength, slice thickness, voxel size, slice gap) may not always be perfectly standardized across patients. However, our results should be relatively unaffected by issues of slice thickness, image quality, and voxel size, since we decided to analyze a set of volumetric features rather than measurements that would be more directly influenced by these subtle differences. Since the majority of the tumors were relatively large, and orders of magnitude larger than the size of an individual voxel (even accounting for voxel variability), similar to other papers published using this dataset, we therefore believe our conclusions are not largely influence by such factors. We should also note that there is no clear association between contributing site, and at least within a site the scanner(s) used were much more consistent than between contributing sites.

In conclusion, our results show that GBM mutations drive observable phenotypes that are quantifiable with MRI imaging. We demonstrate that somatic mutations are associated with macroscopic characteristics and that these clinically important mutations can be significantly predicted with high performance. These results may impact personalized medicine, as imaging is noninvasive and already applied routinely in clinical practice throughout a course of treatment. Finally, our results may shed insights into unique behavioral and macroscopically visible growth characteristics of individual tumors as a result of tumor mutational differences.

## Electronic supplementary material

ESM 1 (DOCX 38 kb)
