# Supplementary material for: Somatic mutations associated with MRI-derived volumetric features in glioblastoma
Source: Neuroradiology. 2015 Sep 4;57(12):1227–37. doi: 10.1007/s00234-015-1576-7 (PMC4648958; doi:10.1007/s00234-015-1576-7)
Supplement: Supplementary file 1 — (DOCX 38 kb) [file 234_2015_1576_MOESM1_ESM.docx]

**ELECTRONIC SUPPLEMENTARY MATERIAL**

Somatic mutations associated with MRI Derived Volumetric Features in Glioblastoma

David A. Gutman, MD PhD^1,2^, William D. Dunn Jr, B.S.^1,2^, Patrick Grossmann, M.Sc.^5^, Lee A.D. Cooper, PhD^2,4^, Chad A. Holder^3^, MD, Keith L. Ligon, MD PhD*^8^*, Brian M. Alexander, MD MPH*^5^*, Hugo J.W.L. Aerts, PhD^5,6,7^

*Departments of Neurology^1^, Biomedical Informatics^2^, Radiology and Imaging Sciences^3^, Emory University School of Medicine, Atlanta, GA, USA.*

*Department of Biomedical Engineering^4^, Georgia Institute of Technology, Atlanta, GA, USA.*

*Departments of Radiation Oncology^5^, Radiology^6^, Biostatistics & Computational Biology^7^, Pathology^8^, Dana-Farber Cancer Institute, Brigham and Women’s Hospital, Harvard Medical School, Boston, MA, USA.*

CORRESPONDING AUTHOR:

David Gutman, MD PhD

Department of Biomedical Informatics

Emory University School of Medicine

Email: [DGutman@emory.edu](mailto:DGutman@emory.edu)

|  |  | **TP53** | **PTEN** | **NF1** | **EGFR** | **IDH1** | **PIK3CA** | **RB1** | **PIK3R1** | **PDGFRA** |
| --- | --- | --- | --- | --- | --- | --- | --- | --- | --- | --- |
|  | **Number of mutations:** | **26** | **23** | **9** | **24** | **5** | **11** | **8** | **12** | **6** |
| Contrast Enhancement | Mut – WT | -8588.05 | 228.07 | 8482.33 | 818.21 | -5163.39 | -1541.38 | -3676.05 | -1522.22 | 6888.52 |
|  | p-value | **0.012*** | 0.955 | 0.143 | 0.827 | 0.403 | 0.745 | 0.485 | 0.774 | 0.359 |
| Necrosis | Mut – WT | -7159.16 | 180.69 | 6758.20 | 3555.12 | -5445.24 | -3537.73 | -4436.73 | 94.53 | 2377.99 |
|  | p-value | **0.017*** | 0.961 | 0.186 | 0.303 | 0.246 | 0.367 | 0.289 | 0.983 | 0.657 |
| T2-FLAIR hyperintensity | Mut – WT | -11164.70 | -13022.60 | -4222.98 | 6655.63 | 1867.0020 | 2754.26 | -26354.40 | -6266.53 | -3245.31 |
|  | p-value | 0.326 | 0.235 | 0.551 | 0.538 | 0.931 | 0.87 | **0.015*** | 0.672 | 0.899 |
| Tumor Bulk | Mut – WT | -15747.20 | 408.77 | 15240.54 | 4373.33 | -10608.60 | -5079.11 | -8112.78 | -1427.70 | 9266.50 |
|  | p-value | **0.012*** | 0.957 | 0.157 | 0.533 | 0.323 | 0.546 | 0.387 | 0.88 | 0.458 |
| Total Tumor | Mut – WT | -26911.90 | -12613.80 | 11017.56 | 11028.92 | -8741.62 | -2324.80 | -34467.20 | -7694.25 | 6021.19 |
|  | p-value | **0.04*** | 0.336 | 0.389 | 0.402 | 0.612 | 0.901 | **0.02*** | 0.611 | 0.858 |
| Necrosis / Contrast Enhancement | Mut – WT | -0.049 | -0.074 | -0.037 | 0.14 | -0.064 | 0.061 | -0.14 | 0.11 | -0.12 |
|  | p-value | 0.492 | 0.254 | 0.587 | **0.05*** | 0.351 | 0.696 | 0.056 | 0.313 | 0.248 |
| Contrast Enhancement / Tumor Bulk | Mut – WT | 0.012 | 0.017 | 0.0017 | -0.046 | 0.0091 | -0.0038 | 0.037 | -0.032 | 0.034 |
|  | p-value | 0.515 | 0.353 | 0.929 | **0.008*** | 0.659 | 0.912 | 0.145 | 0.184 | 0.339 |
| Contrast Enhancement / Total Tumor | Mut – WT | -0.033 | -0.011 | -0.0021 | -0.070 | -0.046 | -0.016 | 0.015 | 0.099 | 0.10 |
|  | p-value | 0.51 | 0.809 | 0.957 | 0.112 | 0.551 | 0.757 | 0.804 | 0.526 | 0.241 |
| Necrosis / Total Tumor | Mut – WT | -0.039 | -0.017 | -0.0057 | -0.030 | -0.045 | -0.037 | -0.015 | 0.13 | 0.025 |
|  | p-value | 0.395 | 0.706 | 0.878 | 0.479 | 0.506 | 0.422 | 0.754 | 0.404 | 0.573 |
| T2-FLAIR hyperintensity/ Total Tumor | Mut – WT | 0.072 | 0.028 | 0.0078 | 0.10 | 0.091 | 0.053 | -0.00012 | -0.23 | -0.13 |
|  | p-value | 0.444 | 0.755 | 0.917 | 0.239 | 0.527 | 0.584 | 0.999 | 0.461 | 0.292 |
| Tumor Bulk/ Total Tumor | Mut – WT | -0.072 | -0.028 | -0.0078 | -0.10 | -0.091 | -0.053 | 0.00013 | 0.23 | 0.13 |
|  | p-value | 0.444 | 0.755 | 0.917 | 0.239 | 0.527 | 0.584 | 0.999 | 0.461 | 0.292 |
|  |  |  |  |  |  |  |  |  |  |  |

**Supplement Table 1:** Differences in imaging feature volumes between mutated and wildtype tumors for all analyzed genes and imaging features. “Mut-WT” refers to difference in average between mutated and wild-type groups for the various volumes (in mm^3^) as well as differences in ratios. For each gene and imaging feature, a two-sided student t-test was performed to measure significance of the difference and the corresponding *p-*value is also provided. All values in bold with asterisk show statistical significance (p < 0.05).

|  |  | **TP53** | **PTEN** | **NF1** | **EGFR** | **IDH1** | **PIK3CA** | **RB1** | **PIK3R1** | **PDGFRA** |
| --- | --- | --- | --- | --- | --- | --- | --- | --- | --- | --- |
|  | **Number of mutations:** | **26** | **23** | **9** | **24** | **5** | **11** | **8** | **12** | **6** |
| Contrast Enhancement | AUC | 0.679 | 0.513 | 0.681 | 0.503 | 0.6 | 0.516 | 0.57 | 0.547 | 0.621 |
|  | p-value | **0.001*** | 0.861 | **0.023*** | 0.971 | 0.37 | 0.87 | 0.49 | 0.611 | 0.258 |
|  | 95% CI | 0.569-0.788 | 0.37-0.655 | 0.525-0.837 | 0.361-0.644 | 0.382-0.818 | 0.326-0.706 | 0.372-0.768 | 0.366-0.727 | 0.411-0.831 |
| Necrosis | AUC | 0.666 | 0.537 | 0.658 | 0.556 | 0.611 | 0.579 | 0.588 | 0.501 | 0.572 |
|  | p-value | **0.004*** | 0.601 | 0.063 | 0.43 | 0.247 | 0.351 | 0.365 | 0.988 | 0.531 |
|  | 95% CI | 0.552-0.78 | 0.397-0.677 | 0.491-0.825 | 0.417-0.695 | 0.423-0.8 | 0.413-0.746 | 0.397-0.779 | 0.329-0.673 | 0.347-0.796 |
| T2-FLAIR hyperintensity | AUC | 0.591 | 0.581 | 0.537 | 0.542 | 0.53 | 0.508 | 0.66 | 0.531 | 0.56 |
|  | p-value | 0.127 | 0.226 | 0.479 | 0.499 | 0.807 | 0.938 | **0.022*** | 0.709 | 0.687 |
|  | 95% CI | 0.474-0.708 | 0.45-0.712 | 0.435-0.639 | 0.42-0.665 | 0.292-0.767 | 0.318-0.698 | 0.523-0.797 | 0.367-0.695 | 0.268-0.852 |
| Tumor Bulk | AUC | 0.675 | 0.53 | 0.671 | 0.535 | 0.611 | 0.556 | 0.577 | 0.529 | 0.612 |
|  | p-value | **0.002*** | 0.675 | **0.032*** | 0.632 | 0.269 | 0.53 | 0.441 | 0.746 | 0.322 |
|  | 95% CI | 0.566-0.785 | 0.39-0.67 | 0.514-0.828 | 0.393-0.676 | 0.414-0.809 | 0.381-0.73 | 0.381-0.773 | 0.355-0.702 | 0.39-0.833 |
| Total Tumor | AUC | 0.646 | 0.539 | 0.604 | 0.551 | 0.521 | 0.521 | 0.676 | 0.533 | 0.515 |
|  | p-value | **0.01*** | 0.562 | 0.103 | 0.445 | 0.811 | 0.813 | **0.012*** | 0.673 | 0.92 |
|  | 95% CI | 0.534-0.758 | 0.407-0.671 | 0.479-0.729 | 0.421-0.681 | 0.348-0.694 | 0.344-0.699 | 0.54-0.813 | 0.382-0.684 | 0.218-0.813 |
| Necrosis / Contrast Enhancement | AUC | 0.531 | 0.549 | 0.516 | 0.682 | 0.527 | 0.516 | 0.642 | 0.618 | 0.612 |
|  | p-value | 0.655 | 0.488 | 0.855 | **0.001*** | 0.767 | 0.88 | 0.066 | 0.127 | 0.376 |
|  | 95% CI | 0.397-0.664 | 0.411-0.687 | 0.348-0.683 | 0.571-0.793 | 0.35-0.704 | 0.309-0.722 | 0.491-0.793 | 0.466-0.771 | 0.364-0.859 |
| Contrast Enhancement / Tumor Bulk | AUC | 0.531 | 0.549 | 0.516 | 0.682 | 0.527 | 0.516 | 0.642 | 0.618 | 0.612 |
|  | p-value | 0.655 | 0.488 | 0.855 | **0.001*** | 0.767 | 0.88 | 0.066 | 0.127 | 0.376 |
|  | 95% CI | 0.397-0.664 | 0.411-0.687 | 0.348-0.683 | 0.571-0.793 | 0.35-0.704 | 0.309-0.722 | 0.491-0.793 | 0.466-0.771 | 0.364-0.859 |
| Contrast Enhancement / Total Tumor | AUC | 0.517 | 0.537 | 0.574 | 0.576 | 0.527 | 0.541 | 0.592 | 0.564 | 0.682 |
|  | p-value | 0.807 | 0.612 | 0.279 | 0.248 | 0.856 | 0.68 | 0.434 | 0.517 | 0.074 |
|  | 95% CI | 0.379-0.655 | 0.393-0.681 | 0.44-0.709 | 0.447-0.704 | 0.238-0.815 | 0.347-0.734 | 0.362-0.822 | 0.371-0.757 | 0.482-0.883 |
| Necrosis/ Total Tumor | AUC | 0.526 | 0.532 | 0.592 | 0.515 | 0.566 | 0.53 | 0.544 | 0.507 | 0.661 |
|  | p-value | 0.709 | 0.663 | 0.19 | 0.82 | 0.629 | 0.754 | 0.684 | 0.947 | 0.063 |
|  | 95% CI | 0.391-0.66 | 0.389-0.674 | 0.454-0.731 | 0.387-0.642 | 0.297-0.835 | 0.344-0.715 | 0.332-0.756 | 0.314-0.699 | 0.491-0.831 |
| T2-FLAIR hyperintensity/ Total Tumor | AUC | 0.522 | 0.535 | 0.576 | 0.549 | 0.561 | 0.501 | 0.572 | 0.539 | 0.722 |
|  | p-value | 0.755 | 0.631 | 0.256 | 0.448 | 0.663 | 0.994 | 0.526 | 0.695 | **0.026*** |
|  | 95% CI | 0.383-0.661 | 0.392-0.677 | 0.445-0.707 | 0.422-0.676 | 0.289-0.833 | 0.317-0.685 | 0.35-0.793 | 0.344-0.734 | 0.527-0.918 |
| Tumor Bulk/ Total Tumor | AUC | 0.522 | 0.535 | 0.576 | 0.549 | 0.561 | 0.501 | 0.572 | 0.539 | 0.722 |
|  | p-value | 0.755 | 0.631 | 0.256 | 0.448 | 0.663 | 0.994 | 0.526 | 0.695 | **0.026*** |
|  | 95% CI | 0.383-0.661 | 0.392-0.677 | 0.445-0.707 | 0.422-0.676 | 0.289-0.833 | 0.317-0.685 | 0.35-0.793 | 0.344-0.734 | 0.527-0.918 |
|  |  |  |  |  |  |  |  |  |  |  |

**Supplement Table 2:** Gene mutation / volumetric imaging feature correlations. For each gene and imaging feature, area under the curve (AUC) values, corresponding *p-*value, and 95% confidence interval are provided. All values in bold with asterisk show statistical significance (p < 0.05).
